# Pupil Dilation and the Slow Wave ERP Reflect Surprise about Choice Outcome Resulting from Intrinsic Variability in Decision Confidence

**DOI:** 10.1093/cercor/bhab032

**Published:** 2021-04-05

**Authors:** Jan Willem de Gee, Camile M C Correa, Matthew Weaver, Tobias H Donner, Simon van Gaal

**Affiliations:** Department of Psychology, Amsterdam Brain & Cognition (ABC), University of Amsterdam, Nieuwe Achtergracht 129-B, 1018WS, Amsterdam, the Netherlands; Department of Neurophysiology and Pathophysiology, University Medical Center Hamburg-Eppendorf, Building N43, Martinistraße 52, 20246, Hamburg, Germany; Department of Neuroscience, Baylor College of Medicine, 1 Baylor Plaza, Houston, TX 77030, USA; Jan and Dan Duncan Neurological Research Institute, Texas Children’s Hospital, 1250 Moursund St, Houston, TX 77030, USA; Department of Psychology, Amsterdam Brain & Cognition (ABC), University of Amsterdam, Nieuwe Achtergracht 129-B, 1018WS, Amsterdam, the Netherlands; Centre of Functionally Integrative Neuroscience, Aarhus University, 44 Nørrebrogade Building 1A, 8000 Aarhus, Denmark; Department of Psychology, Amsterdam Brain & Cognition (ABC), University of Amsterdam, Nieuwe Achtergracht 129-B, 1018WS, Amsterdam, the Netherlands; Department of Psychology, Amsterdam Brain & Cognition (ABC), University of Amsterdam, Nieuwe Achtergracht 129-B, 1018WS, Amsterdam, the Netherlands; Department of Neurophysiology and Pathophysiology, University Medical Center Hamburg-Eppendorf, Building N43, Martinistraße 52, 20246, Hamburg, Germany; Department of Psychology, Amsterdam Brain & Cognition (ABC), University of Amsterdam, Nieuwe Achtergracht 129-B, 1018WS, Amsterdam, the Netherlands

**Keywords:** arousal, confidence, consciousness, prediction error, pupil size

## Abstract

Central to human and animal cognition is the ability to learn from feedback in order to optimize future rewards. Such a learning signal might be encoded and broadcasted by the brain’s arousal systems, including the noradrenergic locus coeruleus. Pupil responses and the positive slow wave component of event-related potentials reflect rapid changes in the arousal level of the brain. Here, we ask whether and how these variables may reflect surprise: the mismatch between one’s expectation about being correct and the outcome of a decision, when expectations fluctuate due to internal factors (e.g., engagement). We show that during an elementary decision task in the face of uncertainty both physiological markers of phasic arousal reflect surprise. We further show that pupil responses and slow wave event-related potential are unrelated to each other and that prediction error computations depend on feedback awareness. These results further advance our understanding of the role of central arousal systems in decision-making under uncertainty.

## Introduction

Pupil dilation at constant luminance has been related to deviant (unexpected) stimuli (Raisig et al. 2010; Murphy, Vandekerckhove, et al. 2014b; Kloosterman et al. 2015; Kamp and Donchin 2015; Knapen et al. 2016; Wetzel et al. 2016; Liao et al. 2018; Van Slooten et al. 2018; Alamia et al. 2019; Zhao et al. 2019; Bianco et al. 2020), behavioral error awareness (Critchley 2005; Murphy et al. 2016; Maier et al. 2019), and to key computational variables such as “uncertainty” about the state of the world and/or the right course of action and subsequent “surprise” after finding out (Richer and Beatty 1987; Preuschoff et al. 2011; Nassar et al. 2012; O’Reilly et al. 2013; Lempert et al. 2015; De Berker et al. 2016; Krishnamurthy et al. 2017; Urai et al. 2017; Colizoli et al. 2018; Van Slooten et al. 2018; Findling et al. 2019; Vincent et al. 2019; Murphy et al. 2020; Filipowicz et al. 2020). Pupil size closely tracks fluctuations in the cortical arousal state mediated by subcortical neuromodulatory systemzs like the noradrenergic locus coeruleus and the cholinergic basal forebrain (Aston-Jones and Cohen 2005; McGinley et al. 2015; Larsen and Waters 2018; Joshi and Gold 2020). Indeed, during elementary decisions, the role of these neuromodulatory systems might be to broadcast information about momentary uncertainty and surprise (Aston-Jones and Cohen 2005; Bouret and Sara 2005; Yu and Dayan 2005; Parikh et al. 2007; Doya 2008; Lak et al. 2017), a process that can be read out by monitoring pupil size.

In this literature, uncertainty and surprise depended lawfully on “external” factors, such as the strength of the stimulus that needs to be discriminated or the volatility of the environment. Strikingly, even when we are given the same information to act on and all external factors are held constant, we will often choose differently each time when asked to make a decision (Glimcher 2005; Sugrue et al. 2005; Gold and Shadlen 2007; Wyart and Koechlin 2016). Such repeated decisions also tend to be associated with varying levels of uncertainty (or the inverse: confidence in being correct) (Fleming and Dolan 2012; Fleming and Lau 2014; Meyniel et al. 2015). Choice and confidence variability of this kind must be driven by “internal” variables.

It is unknown if pupil-linked arousal tracks surprise about choice outcome that results from intrinsic variability in decision confidence. This is important because deviations between objective task performance and subjective decision confidence are commonly observed, both in healthy humans and in several pathologies. Furthermore, it is currently unclear how peripheral markers relate to neural markers of surprise. The phasic release of neuromodulators may be captured in the size of different components of the late positive complex, the P3a, P3b, and slow wave event-related potential (ERP) components, as measured with electroencephalography (EEG) (Friedman et al. 1973; Steinhauer and Zubin 1982; Pineda et al. 1989; Nieuwenhuis et al. 2005; Polich 2007; Murphy et al. 2011; Boldt and Yeung 2015; Brown et al. 2015; Kamp and Donchin 2015; Jepma et al. 2016). The late positive complex has been shown to scale with novelty, surprise, and perceptual confidence in previous studies (Yeung and Summerfield 2012; Boldt and Yeung 2015). Finally, it remains an open question if and how surprise also depends on the subjective awareness of the feedback stimulus. Although unconscious stimuli are known affect a plethora of cognitive processes, it is unknown how important feedback awareness is for prediction error computation (van Gaal and Lamme 2012).

To tackle these questions, we combined an elementary perceptual decision paradigm, including explicit confidence ratings and high or low visibility feedback, with simultaneous pupil size and EEG recordings. We found that 1) both feedback-related pupil responses and ERP slow wave amplitudes reflected surprise about decision outcome, 2) the same pupil and ERP amplitudes were unrelated to each other, and 3) surprise about decision outcome, as reflected by the pupil and/or slow wave ERP, depends on the conscious access to the feedback stimulus.

## Materials and Methods

### Subjects

Thirty-two students from the University of Amsterdam (23 women; aged 18–24) participated in the study for course credits or financial compensation. All subjects gave their written informed consent prior to participation, were naive to the purpose of the experiments, and had normal or corrected-to-normal vision. All procedures were executed in compliance with relevant laws and institutional guidelines and were approved by the local ethical committee of the University of Amsterdam.

### Tasks

Subjects participated in three experimental sessions, separated by less than 1 week. We will first explain the main task, performed in sessions 2 and 3, and thereafter the tasks performed in the first session. In each session, subjects were seated in a silent and dark room (dimmed light), with their head positioned on a chin rest, 60 cm in front of the computer screen. The main task was performed while measuring pupil and EEG responses.

#### Main Task: Orientation Discrimination Task (Sessions 2 and 3)

**Figure 1:**
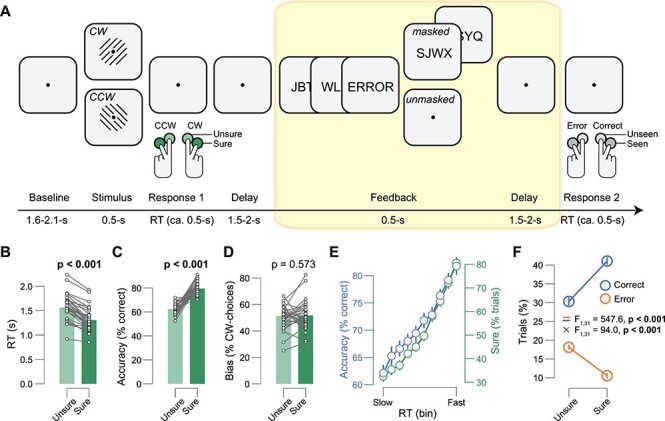
Task and behavior. (*A*) Sequence of events during a single trial. Subjects reported the direction and level of confidence in the decision about a Gabor patch by pressing one of four buttons (CCW = counter-clock-wise, CW = clock-wise; CCW sure; CCW unsure, CW unsure, CW sure). After the decision interval, veridical feedback was presented indicating the correctness of the response. Subjects reported the identity and visibility of the feedback stimulus (the word “error” or “correct” in Dutch) by pressing one of four buttons (seen error; unseen error; unseen correct; seen correct; see Methods for details). (*B*) RT separated for sure versus unsure decisions. Data point, individual subjects; stats, paired-samples *t*-test. (*C*) As *B*, but for accuracy. (*D*) As *B*, but for choice bias. (*E*) Proportion of correct (blue) and sure (green) choices for 10 RT-defined bins. Error bars, SEM. (*F*) Proportion of trials sorted by correctness (error, correct) and confidence (sure, unsure). Stats, ANOVA (Materials and Methods); error bars, SEM. *B*–*F*: Group average (*N* = 32).

Stimuli were presented on a screen with a spatial resolution of 1280 × 720 pixels, run at a vertical refresh rate of 100 Hz. Each trial consisted of the following consecutive intervals (Fig. 1*A*): 1) the baseline interval (1.6–2.1 s); 2) the stimulus interval (0.5 s; interrogation protocol), the start of which was signaled by a tone (0.2 s duration); 3) the response period (terminated by the participant’s response); 4) a delay (uniformly distributed between 1.5 and 2 s); 5) the feedback interval (0.5 s), the start of which was signaled by the occurrence of a tone (0.2 s duration); 6) a delay (uniformly distributed between 1.5 and 2 s); and 7) the feedback identity response period (terminated by the participant’s response).

During Gabor presentation, the luminance across all pixels was kept constant. A sinusoidal grating (1.47 cycles per degree) was presented for the entire stimulus interval. The grating was either tilted 45° (clockwise, CW) or 135° (counterclockwise, CCW). Grating orientation was randomly selected on each trial, under the constraint that it would occur on 50% of the trials within each block of 60 trials. The grating was presented in a Gaussian annulus of 11.4 cm, with a 10.85 degree visual angle (1.47 cycles per degree). Feedback was signaled by the Dutch word “goed” (correct feedback) or the word “fout” (incorrect feedback), from now on referred to as “correct” and “error” feedback. The words were presented for three frames just below fixation. Feedback was either masked, by presenting both forward and backward masks (masks1-masks2-feedback-masks3-masks4) or unmasked, by presenting only forward masks (masks1-masks2-feedback). Each mask consisted of six randomly scrambled letters (without the letters making up the words “goed” or “fout”). Masks’ types were presented two frames each. Feedback type (masked vs. unmasked) was randomly selected on each trial, under the constraint that it would occur on 50% of the trials within each block of 60 trials (Fig. 1*A*).

Throughout the main experiment, the contrast of the Gabor was fixed at the individual threshold level that yielded about 70% correct choices. Each subject’s individual threshold contrast was determined before the main experiment using an adaptive staircase procedure (Quest). The corresponding threshold contrasts yielded a mean accuracy of 70.9% correct (±0.44% standard error of the mean [SEM]) in the main experiment.

Subjects performed between 12 and 17 blocks (distributed over two measurement sessions), yielding a total of 720–1020 trials per participant. Subjects were instructed to report the orientation of the Gabor, and simultaneously their decision confidence in this decision, by pressing one of four response buttons with their left or right index or middle finger—left middle finger: CCW, sure; left index finger: CCW, unsure; right index finger: CW, unsure; right middle finger: CW, sure. Subjects were also instructed to report the identity and visibility of the feedback by pressing one of four response buttons with their left or right index or middle finger—left middle finger: “error,” seen; left index finger: “error,” unseen; right index finger: “correct,” unseen; right middle finger: “correct,” seen. For analyses, we defined high visibility feedback trials as trials on which the feedback was unmasked and subjects reported it as “seen.” We defined low visibility feedback trials as trials on which feedback was masked and subjects reported it as “unseen.”

Note that indeed, the masking procedure revealed two clear categories: On unmasked trials, subjects were 99.82% correct in their discrimination between feedback identity (error/correct) (SEM = 0.05%), whereas in the masked condition they were 71.21% correct (SEM = 1.69%). Note that chance level in the feedback discrimination task is not 50%, because overall Gabor discrimination performance was 70.9% and feedback presentation was veridical. Therefore, subjects may have been able to anticipate the likelihood of being correct/wrong.

#### Passive Viewing Task (Session 1)

In this control task, subjects fixated their gaze at the center of the screen and passively viewed the words “goed” (correct) and “fout” (error), randomly presented for 100 times. Words were presented for three frames (100 Hz refresh rate) and were not masked.

#### Forced-Choice Visibility Task (Session 1)

In this control task, the words “goed” (correct) or “fout” (error) were presented in the same way as in the main experiment (see above), that is, in a masked or unmasked manner (same timings and presence or absence of masks as described above). Subjects were instructed to report the identity of the presented words, by pressing one of two response buttons with their left or right index finger: left, “error”; right, “correct” (the stimulus–response mapping was counterbalanced across trials and was indicated on the screen after each trial). Subjects performed two blocks, yielding a total of 200 trials per participant.

In total, we tested 49 subjects in the first behavioral and eye-tracking session (namely, the passive viewing and forced-choice visibility tasks), considered a pre-screening procedure. We invited 32 subjects to main experiment performed in the second and third sessions. Six subjects did not enter the main experiment due to various reasons (e.g., dropout, extensive blinking [subjectively assessed by the experimenter during the first session, not based on formal analyses]). Of the remaining 43 subjects, the 32 subjects with the lowest discrimination performance score on the forced-choice discrimination task were invited for the second and third sessions (resulting in an accuracy cutoff of >73%). Discrimination performance for the 32 included subjects varied between 49% and 73% correct. Included subjects were on average 98.87% (SEM = 0.02) correct in the unmasked condition and 61.9% (SEM = 0.02) correct in the masked condition. The average percentage of correct responses for masked words exceeded chance-level performance (*t*_31_ = 11.26, *P* < 0.001).

#### Priming Task (Session 1)

In this control task, subjects were instructed to respond as fast and accurately as possible to eight Dutch words, randomly selected out of five of positive (laugh, happiness, peace, love, and fun) and five (death, murder, angry, hate, and war) of negative in valence, by pressing one of two response buttons with their left or right index finger: left, negative; right, positive. Unknown to our subjects, these words were preceded by the masked words “goed” and “fout,” respectively, “correct” and “incorrect,” three frames each before the positive or negative word targets (12 frames each) in 100 Hz refresh rate. This yielded congruent and incongruent trials. Subjects performed six blocks, yielding a total of 480 trials per participant.

### Data Acquisition

The diameter of the left eye’s pupil was tracked at 1000 Hz with an average spatial resolution of 15–30 min arc, using an EyeLink 1000 system (SR Research, Osgoode, Ontario, Canada). EEG data were recorded and sampled at 512 Hz using a BioSemi Active Two system. Sixty-four scalp electrodes were distributed across the scalp according to the 10–20 International system and applied using an elastic electrode cap (Electro-cap International Inc.) Additional electrodes were two electrodes to control for eye movements (left eye, aligned with the pupil, vertically positioned, each referenced to their counterpart), two reference electrodes at the ear lobes to be used as reference, and two electrodes for heartbeat (positioned at the left of the sternum and in the right last intercostal space).

### Data Analysis

#### Eye Data Preprocessing

Periods of blinks and saccades were detected using the manufacturer’s standard algorithms with default settings. The subsequent data analyses were performed using custom-made Python software. The following steps were applied to each pupil recording: 1) linear interpolation of values measured just before and after each identified blink (interpolation time window, from 150 ms before until 150 ms after blink), 2) temporal filtering (third-order Butterworth, low-pass: 10 Hz), 3) removal of pupil responses to blinks and to saccades, by first estimating these responses by means of deconvolution and then removing them from the pupil time series by means of multiple linear regression (Knapen et al. 2016), and 4) conversion to units of modulation (percent signal change) around the mean of the pupil time series from each block.

#### Quantification of Feedback-Evoked Pupillary Responses

**Figure 2:**
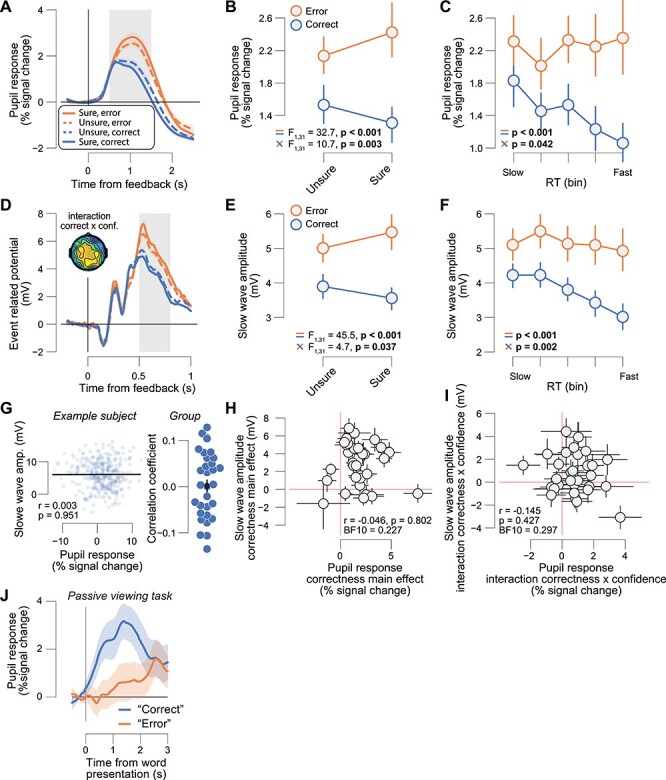
Feedback-related pupil response and slow wave amplitudes report a prediction error. (*A*) High visibility feedback-related pupil time course, sorted by correctness (error, correct) and confidence (sure, unsure). Gray box, interval for averaging pupil response values on single trials. (*B*) High visibility feedback-related pupil responses sorted by correctness (error, correct) and confidence (sure, unsure). Stats, ANOVA (Materials and Methods): Top, correctness main effect; bottom, correctness × confidence interaction. (*C*) High visibility feedback-related pupil responses sorted by correctness (error, correct) and RT. Stats, mixed linear model (Materials and Methods). (*D*), As *A*, but for the high visibility feedback ERP time courses. Head map, correctness × confidence interaction (map limits [−1 1]). (*E*, *F*), as *B*, *C* but for the high visibility feedback-related slow wave amplitudes. (*G*) Left: Trial-by-trial relationship between feedback-related pupil responses and slow wave amplitudes for one example subject. Data points represent single trials. Right: Correlation coefficient of the same relationship separately for each subject. Data points, single subjects (green dots represent significant correlations [*P* < 0.05]); black dot with error bars, group average ± SEM. (*H*) High visibility feedback-related slow wave amplitude correctness main effect ([error/unsure + error/sure] − [correct/unsure + correct/sure]) plotted against feedback-related pupil response correctness main effect. Stats, Pearson correlation; datapoints, individual subjects (*N* = 32); error bars, 60% confidence intervals (bootstrap). (*I*) As *H*, but for correctness × confidence interaction effects ([error/sure − correct/sure] − [error/unsure − correct/unsure]). (*J*) High visibility event-related pupil time course sorted by the words “correct” and “error” during a passive viewing experiment (Materials and Methods). All panels except *H*, *I*: group average (*N* = 32); shading or error bars, SEM.

We computed feedback-evoked pupillary response amplitude measures for each trial as the mean of the pupil size in the window 0.5–1.5 s from feedback, minus the mean pupil size during the 0.5 s before the feedback. This time window was chosen to be centered around the peak of the pupil response to a transient event (like the feedback in our task; Fig. 2*A*) (Hoeks and Levelt 1993; de Gee et al. 2014).

It is commonly observed that task-evoked pupil responses are negatively correlated to pre-trial baseline pupil size (Gilzenrat et al. 2010; de Gee et al. 2014; Mridha et al. 2021; Filipowicz et al. 2020), which is partly due to floor and ceiling effects and a general reversion to the mean. Indeed, pupil responses that occurred time-locked to the decision about Gabor orientation depended negatively on pre-trial baseline pupil size: the group average correlation coefficient (±SEM) was −0.314 (0.015). However, the feedback-related pupil responses (that occurred later in the trial) were not correlated to pre-trial baseline pupil size: The group average correlation coefficient (±SEM) was 0.037 (0.079) in the high visibility condition) and 0.046 (0.061) in the low visibility condition (see Supplementary Figs S1 and S2 for individual subject scatter plots). Therefore, we did not include pre-trial baseline pupil size as a covariate in any of our analyses. All results remain qualitatively the same after including pre-trial baseline pupil size as a covariate (data not shown).

#### E‌EG Data Preprocessing

Standard preprocessing steps were performed in EEGLAB toolbox in Matlab. Data were bandpass filtered from 0.1 to 40 Hz off-line for ERP analyses. Epochs ranging from −1 to 2 s around feedback presentation were extracted. Linear baseline correction was applied to these epochs using a −200- to 0-ms window. The resulting trials were visually inspected and those containing artifacts (e.g., movement artifacts) were removed manually. Moreover, electrodes that consistently contained artifacts were interpolated, entirely or per bad epoch. Finally, using independent component analysis (ICA), artifacts caused by blinks, horizontal eye movements, heartbeats, and single-electrode noise (when excessive only in short periods of time, otherwise the entire electrode was removed and interpolated) were automatically removed from the EEG data using EEGlab. We took a conservative approach and only removed ICA components that were clearly not related to brain activity. On average, 3.95 components were removed per subject.

#### Quantification of Slow Wave Component of the Feedback-Related ERP

We focused on the slow wave component of the feedback-related ERP. The slow wave amplitude on each trial was defined as the mean electrophysiological response in the window 0.5–0.8 s after feedback presentation, measured in a central region of interest (ROI): the averaged signal of electrodes F1, Fz, F2, FCz, FC1, FC2, Cz, C1, C2, CPz, CP1, CP2, Pz, P1, P2. The ROI (electrodes) as well as time window of interest for the single-trial slow waves (0.5–0.8 s) were a priori selected and were identical to a previous study of our group on a similar topic (Correa et al. 2018). ERPs were calculated by taking the mean across all trials. Note that the selection of a ROI in space (electrodes) in combination with a specific time window for the EEG data ensures that the analysis protocol becomes highly similar between pupil and ERP responses. In fact, the same analysis of variance (ANOVA) can be performed with exactly the same factors for both measures (correctness, confidence, visibility), which makes the pupil and ERP results directly comparable and more intuitive to interpret.

For exploratory analysis on the feedback-related negativity (FRN), we used exactly the same ROI as for the slow wave, again identical to a previous study (Correa et al. 2018). The FRN peaked around 400 ms at central electrodes, similar to Correa et al. (2018). We used 350–450 ms post outcome stimulus as our time window of interest for the ANOVA.

#### Behavioral Analyses

Behavioral and statistical analyses were performed in Python. We excluded trials in which a subject blinked during the presentation of the Gabor stimulus (duration, 0.5 s) or the timepoints used to compute feedback-related pupil responses and slow wave amplitudes (0.5–1.5 s from feedback). The group average (± SEM) trial-wise blink rate was 15.43% (2.85%). We only considered unmasked trials reported as seen as high visibility feedback (99% of unmasked trials) and masked trials indicated as unseen as low visibility feedback (89.2% of masked trials). The results are qualitatively the same when using all trials and splitting on seen versus unseen (irrespective of masking) or when using all trials and splitting on masked versus unmasked (irrespective of subjective visibility report) (data not shown). Reaction time (RT) was defined as the time from stimulus offset until the button press.

Autocorrelations in performance might give rise to an artificial correlation between feedback-related pupil responses or slow wave amplitude and behavioral performance on the subsequent trial. Indeed, during the course of an experiment, autocorrelation is typically observed in RTs and accuracy (Gilden 2001; Dutilh et al. 2012; Palva et al. 2013). This could be due to slow drifts in behavioral state factors (e.g., motivation, arousal, attention). We reasoned that a prediction error signal cannot affect performance on the previous trial (because of temporal sequence); instead, any observed relationship must be due to slow autocorrelations in performance. Therefore, in order to isolate the impact of rapid (trial-by-trial) prediction error signal on performance on the next trial from slow ongoing fluctuations, we took the “difference” between the correlation coefficients that captured the trial-by-trial relationship between a prediction error signal (pupil or slow wave amplitude) and performance on the next trial versus the previous. A similar approach has been used before (Desender et al. 2019).

#### Statistical Comparisons

We used 2 × 2 repeated measures ANOVA to test for the main effect of being correct and for the interaction effect between correctness and confidence. With a 2 × 2 × 2 repeated measures ANOVA, we tested whether these main and interaction effects were different between the high and low visibility conditions. We used mixed regression modeling to quantify the trial-by-trial statistical dependence of feedback-related pupil responses or slow wave amplitudes on RT and accuracy. Error variance caused by between-subject differences was accounted for by adding random slopes to the model. Random slopes for a given factor (RT or accuracy) were added only when this increased the model fit, as assessed by model comparison using Bayesian information criterion. We used Pearson correlation to quantify the within- and across-subject correlations. We used the paired-samples *t*-test to test for differences in RT, accuracy, or choices between sure and unsure trials and between congruent and incongruent priming conditions.

#### Data and Code Sharing

The data are publicly available on https://doi.org/10.6084/m9.figshare.14046374. Analysis scripts are publicly available on https://github.com/jwdegee/2021_cereb_cortex_prediction_error.

## Results

### A Prediction Error Signature in Behavior

During simultaneous pupillometry and EEG recordings, 32 human subjects performed a challenging contrast orientation discrimination task (three experimental sessions per subject, on different days). On each trial, this involved discriminating the orientation (CW vs. CCW) of a low-contrast Gabor, explicit confidence ratings, and feedback (Fig. 1*A*). The Gabor’s contrast was adjusted individually such that each subject performed at about 70% correct (Fig. 1*C*; Materials and Methods). Subjects simultaneously indicated their CW versus CCW-choice and the accompanying confidence in that decision (sure vs. unsure; type 1 confidence; Galvin et al. 2003, see Fig. 1*A*). These explicit ratings provided a window into the trial-to-trial fluctuations of decision confidence, which may shape prediction error signals after decision outcome (feedback), and physiological correlates thereof. The Dutch words for “error” or “correct” provided feedback about the correctness of the preceding CW versus CCW-choice. The feedback was masked by random letters on 50% of trials. This was done to manipulate feedback awareness, which may in turn affect uncertainty (about feedback valence) and phasic measures of central arousal state. At the end of the trial, subjects had to indicate the subjective visibility and identity (the word “error” or “correct”) of the feedback stimulus (Fig. 1*A*). This allowed us to post hoc sort trials based on the combination of masking strength and subjective visibility (Materials and Methods).

Subjects’ choice behavior indicated that they successfully introspected perceptual performance: Subjects were faster and more accurate when they were confident in their decision (Fig. 1*B*,*C*), a typical signature of confidence (Meyniel et al. 2013; Kamp and Donchin 2015). There was no relationship between confidence and decision bias (Fig. 1*D*). In line with earlier work (Sanders et al. 2016; Urai et al. 2017), RTs predicted accuracy and confidence, with more accurate and confident choices for faster RTs (Fig. 1*E*). Taken together, these results suggest that subjects in our task were able to introspect perceptual performance well.

Negative feedback (“error”) was more surprising than positive feedback (“correct”), because subjects performed well above chance (~71% correct). Negative feedback should be especially surprising when subjects were relatively sure about the correctness of the preceding choice. In contrast, positive feedback should be least surprising when they were relatively sure about the correctness of the preceding choice. In line with this intuition, trial counts followed the expected ordering (from least to most often, i.e., from most to least surprising): sure/error, unsure/error, unsure/correct, sure/correct (Fig. 1*F*). For trial counts, there was thus a significant main effect for correctness ([error/unsure + error/sure] − [correct/unsure + correct/sure]) and an interaction effect between correctness and confidence ([error/sure − correct/sure] − [error/unsure − correct/unsure]); Fig. 1*F*). Any physiological variable that encodes a prediction error (surprise about decision outcome) should follow a similar pattern. The main effect of correctness may partly reflect error monitoring (Cohen et al. 2011; Ullsperger et al. 2014). Since error monitoring can be triggered purely by the type of feedback received (error vs. correct), we consider especially the interaction between confidence and correctness a signature of a prediction error.

### A Feedback-Related Pupil Response and the Slow-Wave Component of the Feedback-Related ERP Report a Prediction Error

We tested whether feedback-related pupil responses reflect a prediction error after clearly visible feedback. Using the same 2 × 2 ANOVA logic, we found that indeed feedback-related pupil responses (to high visibility feedback) were larger for error versus correct feedback (main effect of correctness) and that this error versus correct difference was larger when subjects were sure versus unsure (interaction correctness × confidence; Fig. 2*A*,*B*). The slow wave component of the feedback-related ERP exhibited a similar functional profile: Slow wave amplitudes were larger after error versus correct feedback and this effect interacted with decision confidence (Fig. 2*D*,*E*; see head map in Fig. 2*D* for a topographical distribution of the interaction between confidence and correctness).

We used the RTs, a sensitive implicit measure of confidence (Fig. 1*E*), to visualize and quantify the pupil- and slow wave-reported prediction errors in a more fine-grained fashion (Urai et al. 2017; Braun et al. 2018). With a mixed linear model, we quantified the trial-by-trial dependence of the feedback-related pupil response on type of feedback (correct vs. error), RT, and their interaction (Materials and Methods). The feedback-related pupil responses were larger for error compared with correct feedback, and this effect interacted with RT (Fig. 2*C*). Likewise, the feedback-related slow wave amplitudes were larger for error compared with correct feedback, and this effect interacted with RT (Fig. 2*F*).

One influential account (Nieuwenhuis et al. 2011) postulates that the pupil response and the slow wave component of the ERP (P3) are driven by the same central (e.g., neuromodulatory) process. This would predict that both measures not only exhibit the same functional profile on average (Fig. 2*A*–*F*), but that they are also correlated at the single-trial level within subjects, and that the magnitude of their respective main and interaction effects is correlated across subjects. Our data did not provide any evidence for those associations: Feedback-related pupil responses and slow wave amplitudes were not correlated at the single-trial level within subjects (Fig. 2*G*, group average *r* = 0.001, SEM = 0.013), and the magnitude of their respective main and interaction effects was not correlated across subjects (Fig. 2*H*,*I*). This suggests that pupil responses and the slow wave component of ERPs are driven by distinct neural processes, both of which are sensitive to decision confidence and prediction errors.

The subject-wise correctness main effect and correctness × confidence interaction effect of feedback-related pupil responses and slow wave amplitudes were not correlated to subjects’ mean accuracy, mean confidence, or metacognitive sensitivity (meta-d’; Verbruggen and Logan 2009; Maniscalco and Lau 2012) (Supplementary Fig. S3).

We verified that the correctness main effect was not driven by any low-level stimulus characteristics, such as luminance, or the intrinsic valence of the words used as feedback (e.g., being of positive/negative valence; Materials and Methods). To that end, before the main experiment, subjects passively viewed the same feedback stimuli while we measured their pupil size (Materials and Methods). In this passive context, the pupil dilated more after the word “correct” compared with “error” (Fig. 2*J*), which is the opposite of what we found in the main experiment (Fig. 2*A*).

The ERP analyses reported so far were performed on the slow wave ERP component (500–800 ms after feedback). We additionally explored the FRN, a frontocentral negative ERP component associated with choice outcome processing and prediction error computation (Cohen et al. 2011; Correa et al. 2018). The FRN can be observed as a small negative difference for the contrast error minus correct feedback, peaking around 400 ms (Fig. 2*D*; see Correa et al. 2018 for a similar timing). Indeed, the FRN was robust after high visibility feedback; however, this effect did not interact with confidence (Supplementary Fig. S4*A*,*B*). Finally, we also zoomed in on the peak of the P3 ERP component (500–600 ms after feedback), and the effects were similar to those for the slow wave component (Supplementary Fig. S4A,C).

Taken together, we conclude that both physiological variables, feedback-related pupil responses and the slow wave component of feedback-related ERPs (including the P3), report a prediction error, when feedback is presented fully consciously.

### Physiological Correlates of Prediction Errors Depend on Feedback Awareness

**Figure 3:**
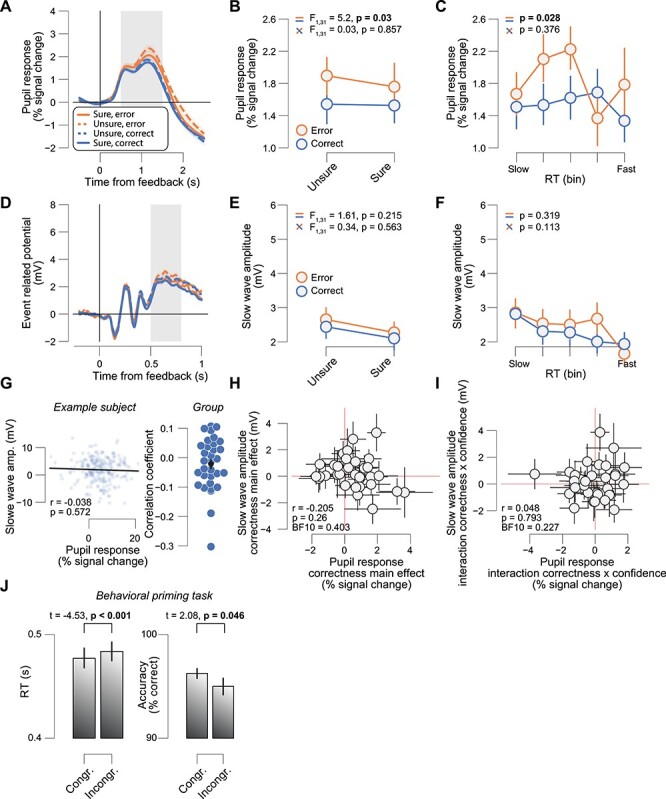
Physiological correlates of prediction errors depend on feedback awareness. (*A*) Low visibility feedback-related pupil time course, sorted by correctness (error, correct) and confidence (sure, unsure). Gray box, interval for averaging pupil response values on single trials. (*B*) Low visibility feedback-related pupil responses sorted by correctness (error, correct) and confidence (sure, unsure). Stats, ANOVA (Materials and Methods): Top, correctness main effect; bottom, correctness × confidence interaction. (*C*) Low visibility feedback-related pupil responses sorted by correctness (error, correct) and RT. Stats, mixed linear model (Materials and Methods). (*D*), As *A*, but for the low visibility feedback ERP time courses. Head map, correctness × confidence interaction (map limits [−1 1]). (*E*, *F*), as *B*, *C* but for the low visibility feedback-related slow wave amplitudes. (*G*) Left: Trial-by-trial relationship between feedback-related pupil responses and slow wave amplitudes for one example subject. Data points represent single trials. Right: Correlation coefficient of the same relationship separately for each subject. Data points, single subjects (green dots represent significant correlations [*P* < 0.05]); black dot with error bars, group average ± SEM. (*H*) Low visibility feedback-related slow wave amplitude correctness main effect ([error/unsure + error/sure] − [correct/unsure + correct/sure]) plotted against feedback-related pupil response correctness main effect. Stats, Pearson correlation; datapoints, individual subjects (*N* = 32); error bars, 60% confidence intervals (bootstrap). (*I*) As *H*, but for correctness × confidence interaction effects ([error/sure − correct/sure] − [error/unsure − correct/unsure]). (*J*) RTs (left) and accuracy (right) sorted by congruency (congruent, incongruent) showing typical behavioral priming effects (Materials and Methods). All panels except *H*, *I*: group average (*N* = 32); error bars, SEM.

We tested whether feedback-related pupil responses and the slow wave component of feedback-related ERPs also report a prediction error after low visibility feedback (Materials and Methods). We did not find evidence for this. For the feedback-related pupil responses, there was a significant correctness main effect, but no correctness × confidence interaction effect (Fig. 3*A*,*B*). For the feedback-related slow wave amplitudes, there was no significant main effect of correctness or an interaction effect thereof with confidence Fig. 3*D*,*E*). The same was true for the FRN and for the peak of the P3 ERP component (Supplementary Fig. S5).

We did also not find evidence for a prediction error using the more fine-grained framework based on RTs. Again, for the feedback-related pupil responses, there was a significant effect of feedback type (correct vs. error), but no interaction effect thereof with RT (Fig. 3*C*). For the feedback-related slow wave amplitudes, there was no significant effect of feedback type or an interaction effect thereof with RT (Fig. 3*F*).

As before, both physiological measures were not correlated at the single-trial level within subjects (Fig. 3*G*, group average *r* = −0.021, SEM = 0.016), and the magnitude of their respective main and interaction effects was not correlated across subjects (Fig. 3*H*,*I*).

We verified that the low visibility feedback was not too weak (because of masking) to drive a potential prediction error. To that end, before the main experiment, subjects completed a behavioral priming experiment with the same stimuli and stimulus timings (Materials and Methods). Subject showed typical priming effects: They were faster and more accurate for congruent prime-target pairs versus incongruent pairs (Fig. 3*J*). Thus, the low visibility feedback was not too weak to potentially drive a prediction error.

Feedback-related pupil responses and slow wave amplitudes reported prediction errors significantly better after high versus low visibility feedback. For the feedback-related pupil responses, there was a significant visibility × correctness interaction effect (*F*_1,31_ = 11.02, *P* = 0.002) and a visibility × correctness × confidence interaction effect (*F*_1,31_ = 6.13, *P* = 0.020). Likewise, for the feedback-related slow wave amplitudes, there was a significant visibility × correctness interaction effect (*F*_1,31_ = 34.73, *P* < 0.001). However, there was no significant visibility × correctness × confidence interaction effect (*F*_1,31_ = 2.67, *P* = 0.112).

In sum, feedback-related pupil responses and the slow wave component of feedback-related ERPs only reflected a prediction error after high visibility feedback (interaction correctness × confidence). Our results indicate that full visibility of decision feedback is critical to drive a prediction error response.

### High Visibility Feedback-Related Slow-Wave Amplitude, but Not Pupil Response, Predicts Speed–Accuracy Adaptation on the Next Trial

Finally, we explored the impact of the observed prediction error responses (feedback-related pupil or slow wave amplitude) on trial-by-trial adjustments of decision-making. To this end, we quantified the relationship between prediction error responses and performance on the subsequent trial, over and above slow drifts in of those variables across trials (see Materials and Methods for details). A correlation between prediction error responses on one trial and performance on the next trial might reflect slow drifts in each of those variables (across multiple trials) as well as the effect of interest here: a rapid (trial-by-trial) adjustment of subsequent decision-making controlled by the prediction error responses. In contrast, such a correlation between a prediction error response on one trial and performance on the “previous” trial cannot reflect adjustments governed by prediction error responses (because of temporal sequence). Therefore, the “difference” between the above two correlations should isolate the effect of prediction error responses on subsequent performance (see also Desender et al. 2019 for a similar approach), and we used this difference measure as our readout of the functional impact of prediction error responses on subsequent performance (accuracy and RT).

High visibility feedback-related slow wave amplitudes predicted both, slower and more accurate responses on the subsequent trial (bars in Supplementary Fig. S6C,D), a hallmark of behavioral speed–accuracy adaptation (Dutilh et al. 2012; Cohen and Van Gaal 2013; Heitz 2014; Desender et al. 2019). These effects were not significant for low visibility feedback (data not shown). By contrast, we found no effect of high visibility feedback-related pupil responses on subsequent performance (bars in Supplementary Fig. S6A,B). High visibility feedback-related slow wave amplitudes did not predict subsequent effects on confidence (group average ∆*r* = −0.126, *P* = 0.161), choice bias (group average ∆*r* = 0.145, *P* = 0.278), or choice repetition probability (group average ∆*r* = −0.217, *P* = 0.089). The same was true for low visibility feedback-related slow wave amplitudes (group average ∆*r* = 0.023, *P* = 0.808; group average ∆*r* = 0.042, *P* = 0.911; group average ∆*r* = −0.116, *P* = 0.443), high visibility feedback-related pupil responses (group average ∆*r* = 0.004, *P* = 1.0; group average ∆*r* = −0.01, *P* = 0.97; group average ∆*r* = 0.068, *P* = 0.513), and low visibility feedback-related pupil responses (group average ∆*r* = 0.085, *P* = 0.369; group average ∆*r* = 0.141, *P* = 0.34; group average ∆*r* = −0.079, *P* = 0.369). In sum, high visibility feedback-related slow wave amplitude, but not pupil response magnitude, predicts speed–accuracy adaptation on the next trial.

## Discussion

Pupil responses and the positive slow wave component of ERPs reflect rapid changes in the arousal level of the brain. We investigated whether and how these variables reflect surprise: the mismatch between one’s expectation about being correct and the outcome of a decision, when expectations fluctuate due to internal factors (e.g., engagement). We show that in an elementary decision-task, feedback-related pupil responses and the slow wave component of feedback-related ERPs reflect surprise. We further show that, within and across subjects, pupil responses and the slow wave component of ERPs are unrelated to each other and that prediction error computations depend on feedback awareness. The results could not be explained by any low-level stimulus characteristics, such as luminance, or the intrinsic valence of the words used as feedback (e.g., being of positive/negative valence, Fig. 2*J*). The reported findings advance our current knowledge about how arousal-linked prediction error computations interact with decision confidence and conscious awareness in several important ways.

For the first time (to our knowledge), we reveal that pupil responses and the slow wave component of ERPs reflect a prediction error that results from intrinsic variability in subjective decision confidence (with all external variables held constant, e.g., task difficulty). Previous studies have revealed that pupil dilation reflects decision uncertainty and prediction error computation during perceptual choices (Urai et al. 2017; Colizoli et al. 2018; Joshi and Gold 2020) when task difficulty was manipulated. In these studies, humans performed a random dot motion task incorporating easy and difficult trials depending on the strength of motion coherence. Pupil responses were larger for performance feedback informing that the decision was erroneous versus correct, and this effect was modulated by trial difficulty, in such a way that the pupil dilated most for erroneous decisions based on strong evidence (strong prediction error) and least for correct decisions based on strong evidence (no prediction error). However, several studies have shown that subjective reports of decision confidence do not necessarily track experimental manipulations of task difficulty, for example, because confidence estimations are biased due to individual differences in sensitivity to evidence strength or affective value (Fleming and Lau 2014; Zylberberg et al. 2014; Lebreton et al. 2018). Because we interrogated subjective decision confidence on every single trial, this allowed us to perform post hoc trial sorting based on trial-by-trial fluctuations in confidence under equal task settings. Thereby, we were able to link feedback processing directly to subjective confidence estimations, establishing direct evidence for prediction error computation reported by the pupil and the slow wave component of the ERP.

Our finding that feedback-related pupil responses and the slow wave component of ERPs were uncorrelated within and across subjects is in line with previous studies (Murphy et al. 2011; Hong et al. 2014; Kamp and Donchin 2015; Mückschel et al. 2017; LoTemplio et al. 2020) and suggests that these established markers of phasic arousal are driven by distinct sources. Recent animal studies have revealed a tight coupling between pupil diameter and neural responses in the noradrenergic locus coeruleus (Varazzani et al. 2015; Joshi et al. 2016; Reimer et al. 2016; Liu et al. 2017; Breton-Provencher and Sur 2019), which is supported by recent human functional magnetic resonance imaging studies (Murphy, O’Connell, et al. 2014a; de Gee et al. 2017). However, some of these studies also found unique contributions to pupil size in other subcortical regions, such as the cholinergic basal forebrain, dopaminergic midbrain, and the superior and inferior colliculi (Joshi et al. 2016; Reimer et al. 2016; de Gee et al. 2017; Mridha et al. 2021). Several lines of evidence reinforce a putative link between pupil diameter and the dopamine system, for example, in patients with Parkinson’s disease (Kringelbach et al. 2007; Weinshenker and Schroeder 2007; Manohar and Husain 2015; Varazzani et al. 2015; Mathôt 2018), and although a link between dopamine and the P3 has also been observed in Parkinson’s patients, evidence is more mixed (see e.g., Bertram et al. 2020). The P3 has also been used as an electrophysiological correlate of feedback-evoked phasic catecholamine release in the cortex (Nieuwenhuis et al. 2005; Polich 2007; Rangel-Gomez et al. 2013). Therefore, although these physiological markers of phasic arousal tend to co-occur, they may be driven by (partly) different sources. In line with this, we found that the behavioral impact of feedback-related pupil responses and the slow wave component of ERPs was also distinct: The slow wave ERP predicted behavioral speed–accuracy adaptation on the subsequent trial, which was absent for pupil size (Murphy et al. 2011; Kamp and Donchin 2015; Eckstein et al. 2017; LoTemplio et al. 2020). It is important to note, however, that different sources of noise contribute to pupil and EEG measurements, which might partly explain the absence of correlation between the two described here. For example, 1) participant fatigue, comfort, readiness, head movements, and eye movements all have a major impact on data quality but might differentially impact the EEG versus pupil size signals; 2) common additional sources of noise in EEG data include the cardiac signal (electrocardiogram, ECG) and movement artifacts caused by muscle contraction, for example, in the neck (electromyogram, EMG), while luminance fluctuations, blinks, and “pupillary hippus” contribute to noise in pupillometry data; and 3) the respective acquisition systems introduce their own measurement noise. Understanding the relationship between changes in pupil dilation and the amplitude of the slow wave component and P3 component is an important avenue for future research.

Further, we show that feedback-related pupil responses and the slow wave component of ERPs only reflect surprise when feedback is fully visible. Although there is consensus that some perceptual and cognitive processes may unfold in the absence of awareness, it is highly debated which functions (if any) may need consciousness to emerge (Dehaene and Naccache 2001; Hommel 2007; Kunde et al. 2012; van Gaal et al. 2012). Many perceptual and cognitive processes may partly unfold unconsciously, typically demonstrated in masked priming studies, in which a task-irrelevant unconscious stimulus facilitates responding to a subsequent task-relevant conscious stimulus (Kiesel et al. 2007; Lamme 2010; Kiefer et al. 2011). These “simple” priming effects are typically explained by assuming that the fast feedforward sweep of neural processing is relatively unaffected by masking and is able to unconsciously affect ongoing behavioral responses (Dehaene and Changeux 2011; van Gaal et al. 2012). We also observed here that the same masked stimuli used as feedback in the main experiment (the words error/correct) could induce behavioral priming when presented in the context of a masked priming task (Fig. 3). However, when the same stimuli were used as feedback stimuli in a perceptual decision task, no prediction error responses were observed (correctness × confidence interaction). Speculatively, error detection mechanisms (main effect of correctness) could still be observed when feedback was masked in the current task design (Fig. 3). Although evidence was statistically relatively weak, the main effects of correctness, especially in pupil size, were significant. This may not be overly surprising, because previous studies have shown that error detection mechanisms may unfold (at least partially) in the absence of error awareness (Nieuwenhuis et al. 2001; Overbeek et al. 2005; Cohen et al. 2009; Shalgi 2012; Charles et al. 2013). However, our results revealed more importantly an absence of confidence × correctness interactions on low visibility feedback. This may suggest that to incorporate subjective confidence in feedback-driven prediction error computations, awareness of the decision outcome (feedback) is crucial. This may suggest that prediction error computation cannot rely on feedforward responses alone, in contrast to, for example, masked priming, and requires (bidirectional) interactions (i.e., recurrent processing) between higher-order and lower-order regions, a phenomenon mainly observed when stimuli are presented above the threshold of conscious perception (Dehaene and Changeux 2011; van Gaal and Lamme 2012). Further unraveling the underlying neural processes dissociating “objective error processing” from “prediction error” computation is important for further understanding the potential scope and limits of unconscious information processing.

Although we observed that confidence-associated surprise was only present when the decision outcome (feedback) was presented fully consciously, previous studies have shown that pupil size is sensitive to “implicit” surprise and effort invested in a cognitive task. For example, it has recently been demonstrated that pupil size increases when the level of cognitive effort invested in a (conflict) task is high, even when subjects are not aware of systematic differences in difficulty between conditions (Diede and Bugg 2017). Related, it has been shown recently that when agents are not aware of specific transitional rules in an implicit learning task, both the pupil and central ERP potentials (reminiscent of the mismatch negativity) may still signal surprise when statistical regularities in stimulus transitions are violated (Alamia et al. 2019; see also Meijs et al. 2018). Although intriguing, both tasks can be considered “implicit” (learning/conflict) tasks, because stimuli were always presented fully consciously and subjects were just not aware of differences in the probabilities of occurrence of specific stimuli. Therefore, these effects cannot be directly compared to situation in which stimulus visibility is reduced.

Although the main goal of this study was to test the association between putative measures of central arousal state (pupil response and the slow wave component of ERPs) and prediction error computation, we also explored the same association for other ERP components associated with feedback processing, such as the FRN and the P3 (Cohen et al. 2011; Correa et al. 2018). Previously, using a probabilistic reversal learning task, we observed that the amplitude of the FRN was strongly linked to the signed prediction error variable (“objective prediction error”) derived from reinforcement learning modeling (Correa et al. 2018). This relationship was strongly attenuated when feedback awareness was reduced. Future work is needed to explore in more detail the relationship between different ERP components (e.g., FRN, P3, slow wave ERP) and specific aspects of prediction error computation and how these may be differentially affected by levels of (feedback) awareness.

## Author contributions

J.W.d.G.: conceptualization, data curation, formal analysis, visualization, writing—original draft preparation, and writing—review and editing. C.M.C.C.: conceptualization, investigation, writing—original draft preparation, writing—review and editing, and funding acquisition. M.W.: formal analysis. T.H.D.: conceptualization, writing—review and editing. S.v.G.: conceptualization, writing—original draft preparation, writing—review and editing, supervision, and funding acquisition.

## Funding

Brazilian Science Without Borders program.

## Notes

*Conflict of Interest:* None declared.

## Supplementary Material

Supplementary_information_bhab032
