# Supplementary material for: Pupil Dilation and the Slow Wave ERP Reflect Surprise about Choice Outcome Resulting from Intrinsic Variability in Decision Confidence
Source: Cereb Cortex. 2021 Apr 5;31(7):3565–78. doi: 10.1093/cercor/bhab032 (PMC8196307; doi:10.1093/cercor/bhab032)
Supplement: Supplementary_information_bhab032 [file supplementary_information_bhab032.pdf]

Supplementary information for the article:

Pupil dilation and the slow wave ERP reflect surprise about choice outcome resulting from intrinsic variability in decision confidence

Jan Willem **de Gee**[1,2,3,4*], Camile M.C. **Correa**[1,5*], Matthew **Weaver**[1], Tobias H. **Donner**[1,2], Simon **van Gaal**[1]

[1]Department of Psychology and Amsterdam Brain & Cognition (ABC), University of Amsterdam, Amsterdam, the Netherlands
[2]Department of Neurophysiology and Pathophysiology, University Medical Center Hamburg-Eppendorf, Hamburg, Germany
[3]Department of Neuroscience, Baylor College of Medicine, Houston, TX, USA
[4]Jan and Dan Duncan Neurological Research Institute, Texas Children's Hospital, Houston, TX, USA
[5]Centre of Functionally Integrative Neuroscience, Aarhus University, Aarhus, Denmark

*Equal contribution
Correspondence: jwdegee@gmail.com (JWdG) or s.vangaal@uva.nl (SvG).

Supplementary figures

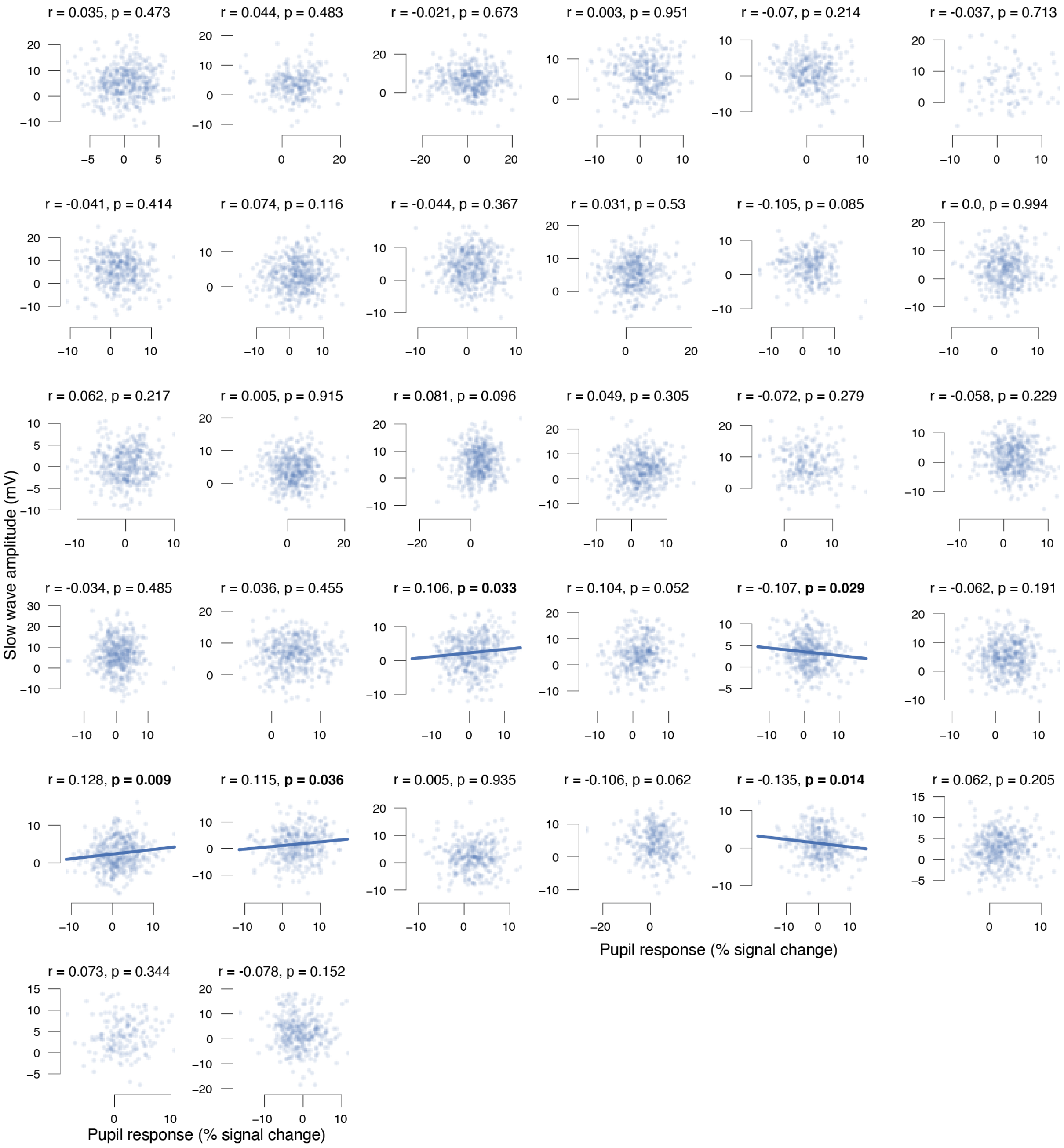

Figure S1. Trial-by-trial relationship between high visibility feedback-related pupil response and pre-trial baseline pupil size. Stats, Pearson correlation; data points, single trials; each panel is a different subject.

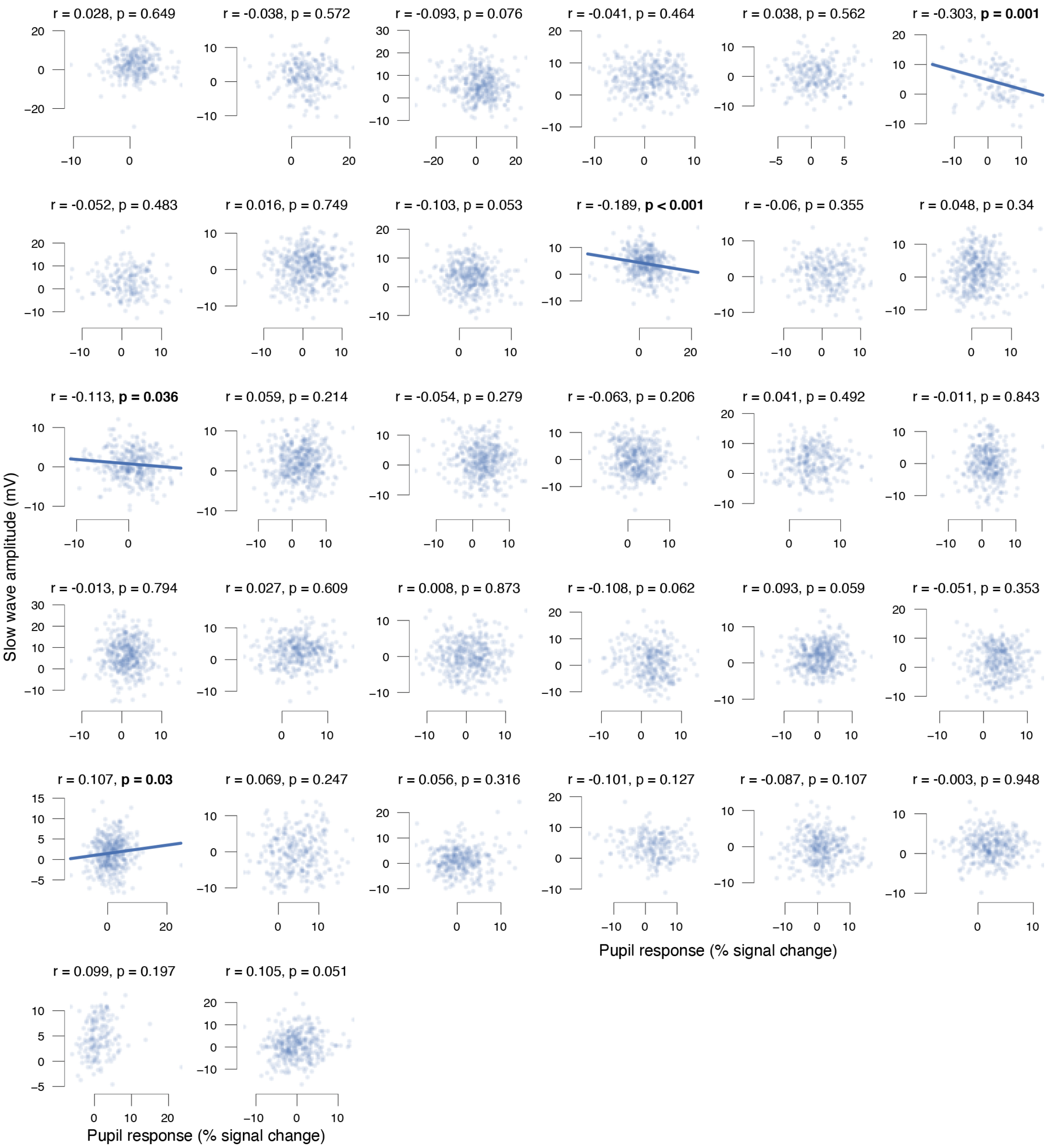

Figure S2. Trial-by-trial relationship between low visibility feedback-related pupil response and pre-trial baseline pupil size. Stats, Pearson correlation; data points, single trials; each panel is a different subject.

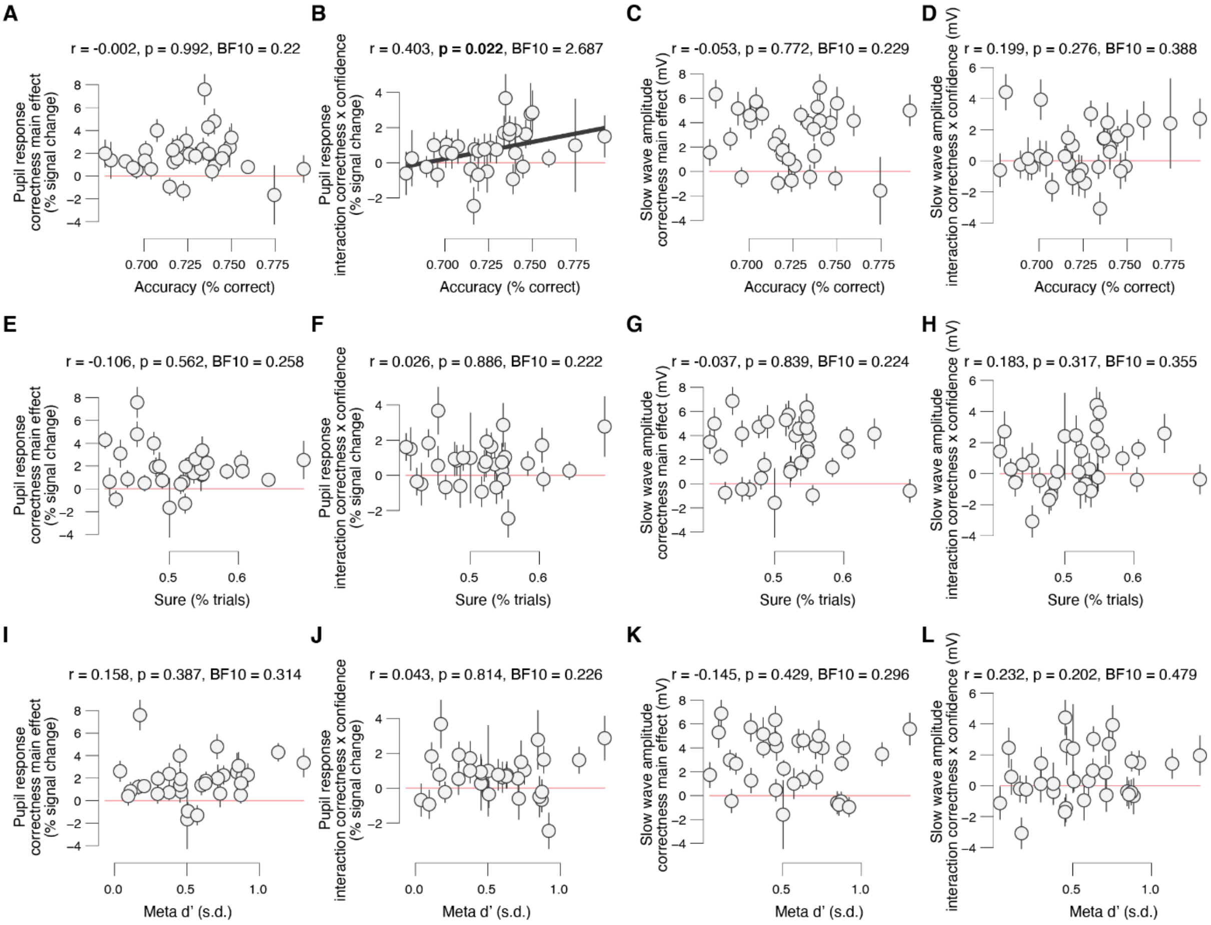

Figure S3. (A) High visibility feedback-related pupil response correctness main effect ([error/unsure + error/sure] - [correct/unsure + correct/sure]) plotted against overall accuracy. Stats, Pearson correlation; datapoints, individual subjects (N=32); error bars, 60% confidence intervals (bootstrap). **(B)** As A, but for correctness x confidence interaction effect ([error/sure - correct/sure] - [error/unsure - correct/unsure]). **(C,D)** As A,B, but for high visibility feedback-related slow wave amplitudes. **(E-F)** As A-D, but for overall confidence. **(I-L)** As A-D, but for overall meta-sensitivity.

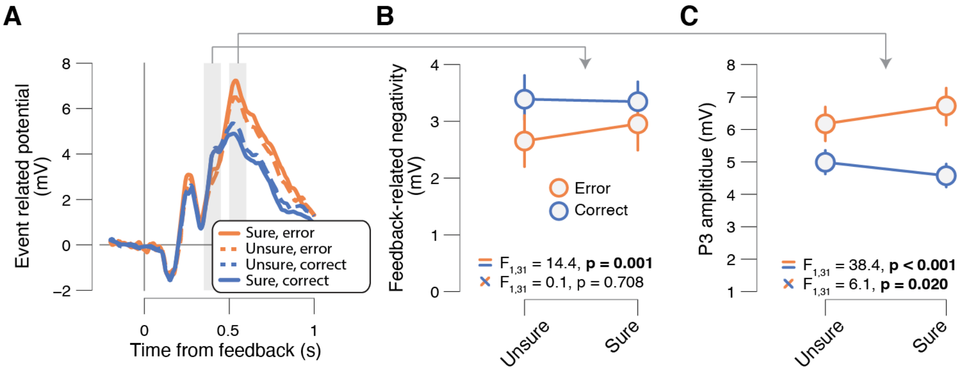

Figure S4. (A) High visibility feedback-related event-related potential (ERP) time courses, sorted by correctness (error, correct) and confidence (sure, unsure). Grey box, interval for averaging values on single trials. **(B)** High visibility feedback-related negativity (FRN) sorted by correctness (error, correct) and confidence (sure, unsure). Stats, ANOVA (Materials and Methods): top, correctness main effect; bottom, correctness x confidence interaction. **(C)** As B, but for P3 peak amplitudes.

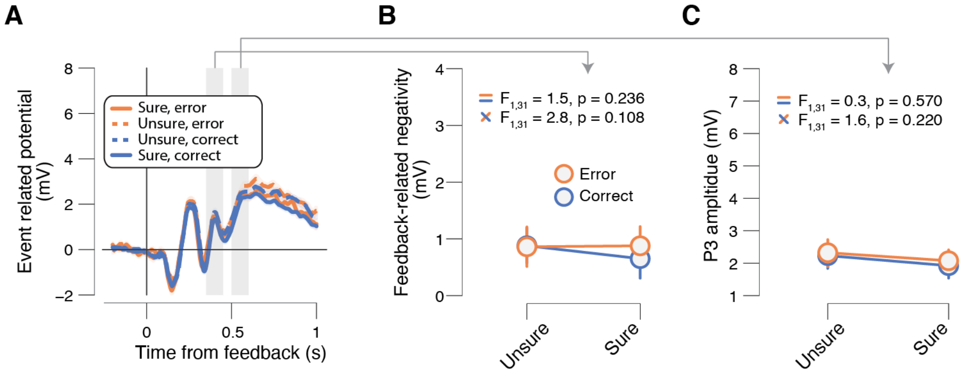

Figure S5. (A) Low visibility feedback-related event-related potential (ERP) time courses, sorted by correctness (error, correct) and confidence (sure, unsure). Grey box, interval for averaging values on single trials. **(B)** Low visibility feedback-related negativity (FRN) sorted by correctness (error, correct) and confidence (sure, unsure). Stats, ANOVA (Materials and Methods): top, correctness main effect; bottom, correctness x confidence interaction. **(C)** As B, but for P3 peak amplitudes.

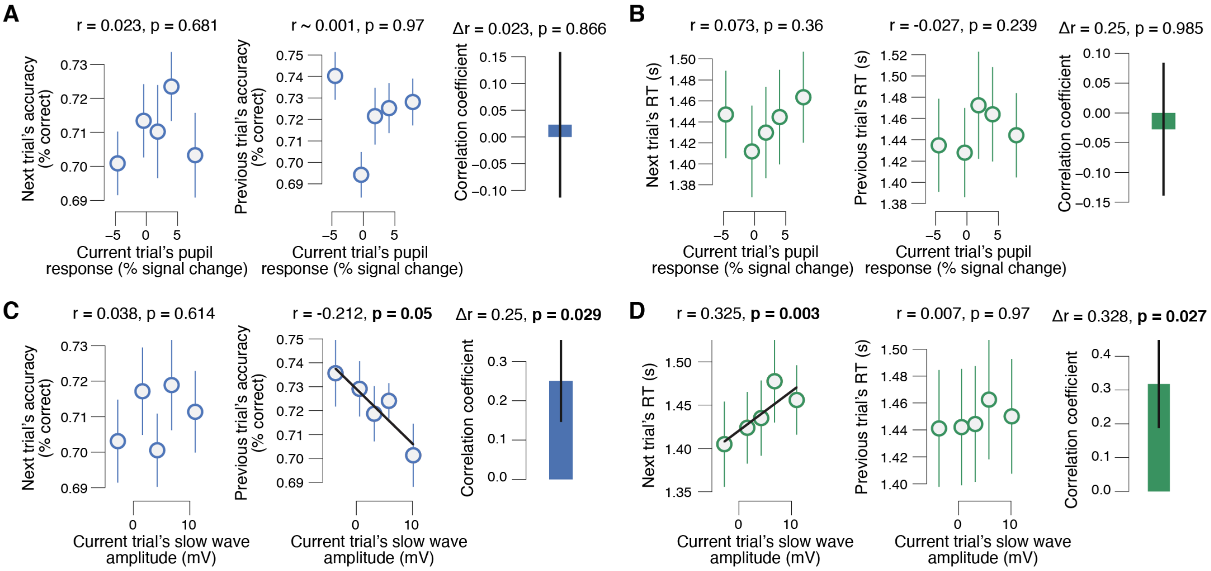

Figure S6. (A) Left: relationship between high visibility feedback-related pupil responses and accuracy on the next trial. Stats, Pearson correlation; error bars, s.e.m. across subjects; data is binned for visualization purposes only. Middle, as left, but for accuracy on the previous trial. Right: group average *difference* between correlation coefficients from left and middle panels. Error bar, s.e.m. across subjects. **(B)** As A, but for RT. **(C,D)** As A,B, but for high visibility feedback-relation slow wave amplitudes.
